# The effect of substrate and product transfer between polydisperse microdroplets on biological high-throughput screens

**DOI:** 10.1038/s41598-025-25878-5

**Published:** 2026-02-02

**Authors:** T. Fecker, T. de Kanter, R. J. van Tatenhove-Pel

**Affiliations:** Department of Biotechnology, Delft University of Technology, van der Maasweg 9, Delft, 2629 HZ The Netherlands

**Keywords:** Droplet encapsulation, Microfluidics, Molecule transfer, High-throughput screening, Emulsions, Biochemistry, Biological techniques, Biotechnology, Chemistry

## Abstract

Microdroplet encapsulation of cells is a powerful tool for high-throughput biological testing. The technique is widely used in mutant selection and strain engineering, where strict compartmentalization is often required to prevent transfer of cells or molecules between droplets. While molecular transfer has been reported for fluorophores, the potential transfer of common small substrates and products, and its impact on biological outcomes, has not been elucidated. Here, using a biosensor system, we demonstrate that biologically relevant molecules such as small alcohols (e.g. ethanol) and weak acids can transfer between droplets. The transfer can alter carbon availability and pH in neighboring droplets. Ethanol, a common product in biological systems, partitions directly into the oil phase, leading to unintended cell growth in adjacent droplets. Weak acids also transfer, decreasing the pH in neighboring droplets. Our results show that the transfer of small, hydrophobic molecules, commonly found in biological systems, can significantly alter experimental outcome. These findings highlight the need for validating droplet compartmentalization in assays involving small, amphiphilic molecules and careful selection of substrates that are not likely to transfer among droplets.

**Supplementary Information:**

The online version contains supplementary material available at 10.1038/s41598-025-25878-5.

## Introduction

Over the last decade, encapsulation of cells in the water droplets of a water-in-oil emulsion has emerged as a powerful tool for meeting the growing demand for high-throughput biological experimentation. Unlike other high-throughput methods like fluorescence-assisted cell sorting (FACS), which require the desired product to be intracellular, droplet encapsulation directly links the producer cell to its environment, making it possible to screen cells with secreted metabolites^1^.

Traditionally, microtiter plate assays have been used for similar screening applications, but their throughput is restricted by plate capacity and liquid handling. In contrast, droplet encapsulation enables cost-effective screening of large parameter spaces, such as mutant libraries, with volumes ranging from femto- to nanolitres^2,3^. This technique has been widely applied in laboratory studies, for example to screen strain libraries to identify variants with elevated protein production^4^, enhanced metabolite secretion^5^, or improved biomass yield^6^.

A key feature of microdroplet systems is their ability to keep the compartments isolated, preventing transfer of substrates or metabolites into neighbouring droplets. To achieve this, researchers mix the aqueous phase containing cells and nutrients with an oil phase, forming an emulsion, stabilized by a surfactant^7^. Polydisperse emulsions (broad size distribution) are easily generated via vortexing or homogenization^8^, while microfluidic chips produce more uniform, monodisperse emulsions^9^. Earlier systems used hydrocarbon-based oils and surfactants, which limited oxygen solubility and allowed for some organic molecules, like ethanol or fluorescein, to transfer into the oil phase^10–12^. To address these issues, fluorinated oil/surfactant systems (using for example HFE7500 as oil phase) have become increasingly popular. Here, most carbon-hydrogen bonds of the oil phase and surfactant are replaced by carbon-fluoride bonds, which reduces interactions with both hydrophilic and hydrophobic molecule groups^10,13^.

Although fluorinated emulsions improve molecule retention in the droplets, recent studies suggest that certain classes of molecules, particularly large, hydrophobic aromatics like fluorophores, can transfer between droplets^14–17^. The molecules transfer at a rate proportional to their hydrophobicity^16^, which is unexpected since organic molecules do not mix well with fluorinated liquids^18^. Since fluorophores often serve as experimental readout, their transfer can compromise experimental accuracy^19^. This issue has prompted researchers to investigate the underlying transfer mechanism^19,20^. Rather than directly diffusing to the neighbouring droplet via the oil phase, these large molecules are often shuttled via micelles formed by excess surfactant molecules, which display an organic head group^17,20,21^. These findings recently sparked an extensive survey of different molecule classes and their ability to transfer between droplets^14^.

Beyond fluorophores, biological microdroplet experiments typically require the strict compartmentalization of small molecules, such as substrates or products. For example, when selecting for high-yield mutants, the available carbon source per cell must be ‘privatized’ in the droplet^6^. However, common metabolic byproducts of biological culturing, such as ethanol, have been suspected to transfer in fluorinated systems^22^. Such transfer can disrupt the strict compartmentalization with potential impact for the neighbouring droplets. For example, ethanol leakage could inhibit growth or provide extra carbon for cells in neighbouring droplets, skewing screening experiments or strain optimization for bioethanol production. For some common molecule classes like amines or organic acids, parameters (e.g., molecule size, hydrophobicity) have been established to assess their potential to transfer^14^. Still, the field lacks a predictive framework to assess the risk of molecule transfer particularly for molecules commonly encountered in biological experiments, covering common substrates and products.

Here, we tested a range of biologically relevant small molecules to evaluate their potential to transfer between droplets in polydisperse systems and the impact on experimental outcomes. We developed a biosensor-based assay to directly test the transfer of relevant substrates, such as sugars, amino acids and alcohols, and small products like organic acids, which can be readily adapted to test other molecules. By monitoring cell growth in adjacent droplets, we quantify the biological effect of small molecule transfer. Further, we test the effect of weak acid transfer on the pH of neighbouring droplets and give a perspective on preventing unwanted transfer. Unlike previous studies, our approach directly links molecular transfer to biological outcomes. Based on our results, we recommend substrates and products suitable for reliable use in droplet-based experiments.

## Results

In water-in-oil (w/o) emulsions, individual cells are compartmentalized in microdroplets of 10–100 μm diameter, enabling rapid screening^7^. The cells are separated from each other by an oil phase, typically a fluorinated oil like HFE7500. To ensure that droplets form independent compartments, neither cells nor substrates should transfer between droplets. To test to what extent commonly encountered molecules disrupt compartmentalization by transferring between droplets and how this transfer impacts compartmentalization in high-throughput droplet screens, we did a series of experiments focusing on common substrates and products.

### Alcohols directly partition into HFE7500 oil

We tested whether a range of substrates and products typically encountered in biological systems partition from the water phase into HFE7500 oil. To do so, we prepared aqueous solutions of sugars, alcohols, and weak acids and mixed them with HFE7500 oil in a 1:9 (aqueous: oil) volumetric ratio. We measured molecule concentrations using HPLC before and after mixing with the oil, to identify whether the molecule partitions into the oil phase. We chose the concentrations of each compound to maximize the signal-to-noise ratio of the HPLC, while maintaining biological relevance. For example, ethanol was chosen to be 180 mM = 8.3 g/L. Typical concentrations for growing *S. cerevisiae* on ethanol are between 3.5 and 7.5 g/L^23^, while aerobic fermentations by *S. cerevisiae* on 20 g/L glucose typically yields 7.5 g/L ethanol^24^, close to the chosen value. Similarly, 0.2–0.4 g/L acetic acid is produced by S. cerevisiae on 20 g/L glucose, close to the chosen tested concentration (10 mM = 0.6 g/L)^25^.

**Fig. 1:**
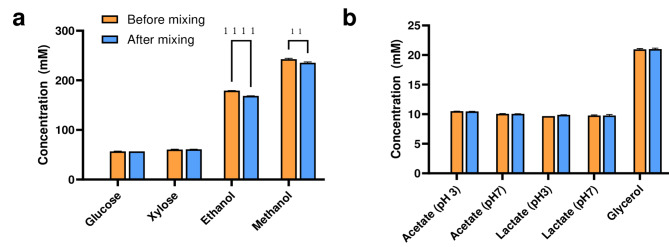
Partitioning of common substrates and products into HFE7500 oil. The concentration of the prepared solution is measured before and after mixing with HFE7500 to give an estimation how much of each molecule partitioned into the oil phase. The bars represent the mean of three replicates ± SD. Asterisks indicate statistically significant differences (α = 0.01) (**: *p* = 0.0033, ****: *p* = 0.000006; unpaired, one-tailed Student’s t-test). (**a**) Sugars and alcohols. (**b**) Weak acids and glycerol. The figure was split for clarity.

While none of the sugars and weak acids showed any loss in concentration, the concentration of the alcohols ethanol and methanol dropped significantly after mixing with HFE7500 oil (6.3 ± 0.3% and 3.0 ± 0.54%, respectively, Fig. 1). This indicates direct partitioning of alcohols into the oil phase, potentially disrupting the compartmentalization of biological systems in microdroplet experiments.

### Ethanol transfer between microdroplets enables growth of *Saccharomyces cerevisiae cells*

To assess the impact of ethanol partitioning on microdroplet compartmentalization, we investigated whether ethanol transfer between droplets could allow for growth of *S. cerevisiae* cells. Polydisperse droplets with a representative volume ± SEM of 37.6 ± 6.9 pL were generated using vortexing (Supplementary Sect. 1, Figure S1). *Saccharomyces cerevisiae* was selected for its ability to grow aerobically on both glucose and ethanol.

**Fig. 2:**
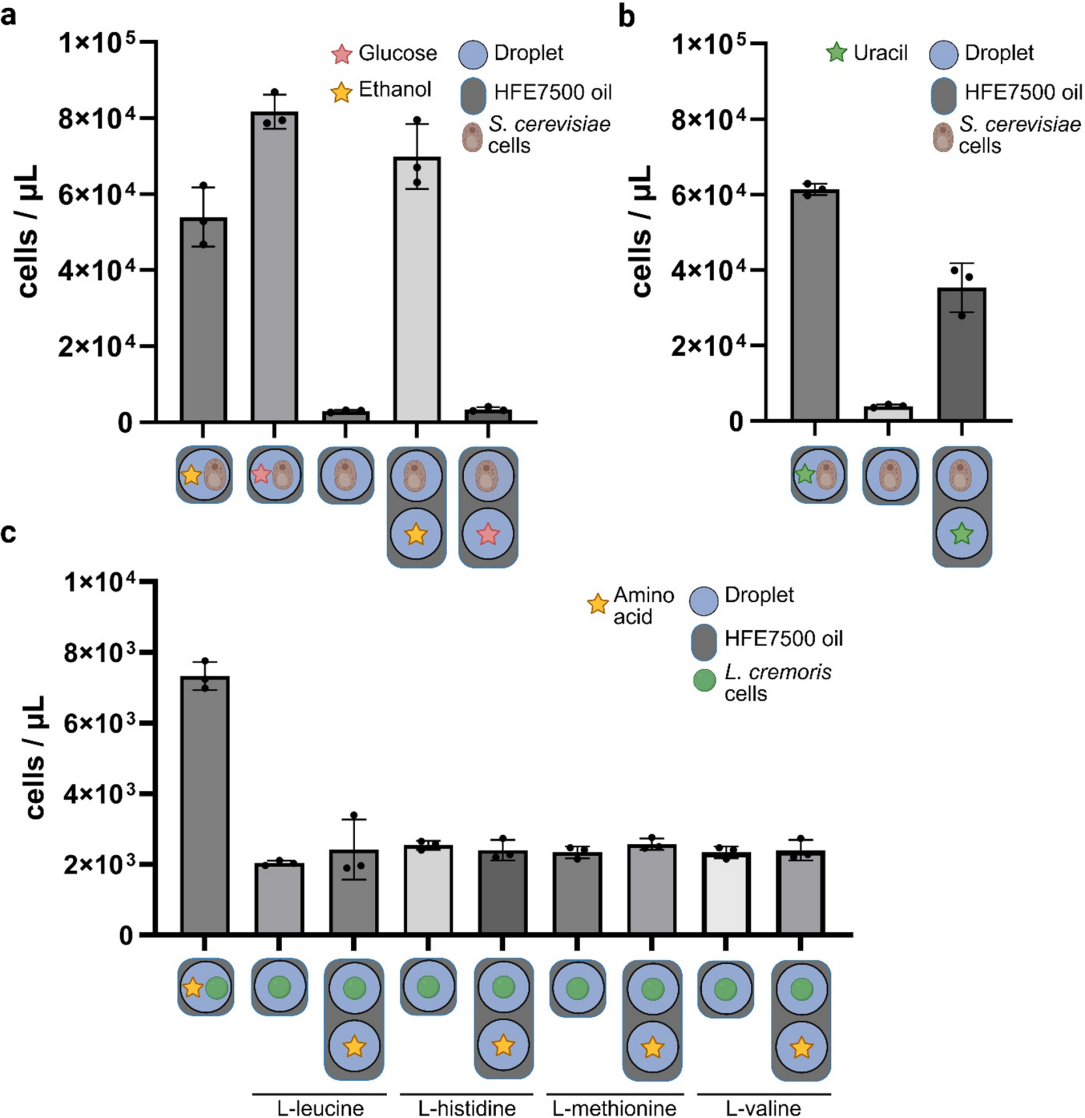
Testing the biological effect of molecule transfer in microdroplets. (a) Molecular transfer of glucose (red stars) and ethanol (yellow stars) in HFE7500 oil. The S. cerevisiae cells were cultured in droplets containing 2 wt% ethanol, 8 wt% glucose or no carbon source, in which case the droplets were mixed with other droplets containing these substrates. Cell growth after 72 h was assessed by breaking the emulsion and subsequent flow cytometry. *n* = 3 mean ± SD. (b) Molecular transfer of uracil in HFE7500 oil. The auxotrophic S. cerevisiae cells were cultured in droplets containing 8 wt% glucose but lacking uracil and mixed with an emulsion containing uracil. Cell growth after 72 h was assessed by breaking the emulsion and subsequent flow cytometry. *n* = 3 mean ± SD (c) Molecular transfer of a selection of amino acids. Auxotrophic L. cremoris cells were grown in four different emulsions, each missing one amino acid and mixed with emulsions containing these amino acids. Cell growth after 72 h was assessed by breaking the emulsions and subsequent flow cytometry *n* = 3 mean ± SD. Figure was created in BioRender. Fecker, T. (2025): https://BioRender.com/bc2n7rs.

We first confirmed that *S. cerevisiae* could grow in microdroplets containing ethanol or glucose, and could not grow in droplets without a carbon source (Fig. 2). To test for carbon source transfer between droplets, we mixed two emulsions – one with cells only and the other one with carbon source only. The results showed restored cell growth in the mixed emulsions containing ethanol, but not in those containing glucose (Fig. 2). This observation indicates that ethanol, unlike glucose, can transfer between microdroplets and induce cell growth.

The emulsions generated containing either glucose or ethanol did not differ significantly in size and size distribution in time (Supplementary Figure S2), indicating that any selective transfer of either substrate could not be explained by differences in microdroplet generation. Additionally, the amount of small droplets (< 2 μm) produced during emulsification and potentially acting as molecular shuttles, did not differ significantly between either emulsion (Supplementary Figure S2), suggesting that the chosen substrate did not impact the droplet generation process.

Next, we tested if other molecule classes would transfer, enabling growth in neighbouring droplets. In this regard, we tested four amino acids (L-leucine, L-valine, L-histidine, and L-methionine), as well as the nucleotide uracil with auxotrophic strains. We chose *L. cremoris* MG1363 as biosensor, as it is auxotrophic for these amino acids^26^, and *S. cerevisiae* CEN.PK113-5D as uracil auxotroph^27^.

While none of the amino acids showed transfer that enabled growth of the auxotrophic *L. cremoris* strain (Fig. 2c), the nucleotide uracil transferred sufficiently to enable growth of the *S. cerevisiae* strain (Fig. 2b).

### Ethanol does not induce a transfer of glucose between microdroplets

In biological experiments with fermenting organisms like *S. cerevisiae*, ethanol is a common product when culturing on glucose. Due to its amphiphilic nature, ethanol has been observed to act as co-solvent, potentially inducing the transfer of molecules by weakening the droplet/oil interface^28^. Co-transfer of ethanol together with glucose would therefore impact strict compartmentalization of glucose.

To investigate whether ethanol could act as a co-solvent for glucose transfer in microdroplets, we employed a system similar as before using *Lactococcus cremoris* as biosensor. The *L. cremoris* strain was chosen for its ability to consume glucose but not ethanol, making it an ideal indicator for glucose transfer in the presence of ethanol. We again prepared two types of emulsions: one containing cells but no carbon source, and another containing either glucose alone or glucose with ethanol. After mixing the emulsions, we incubated the emulsions for 48 h and assessed bacterial growth.

**Fig. 3:**
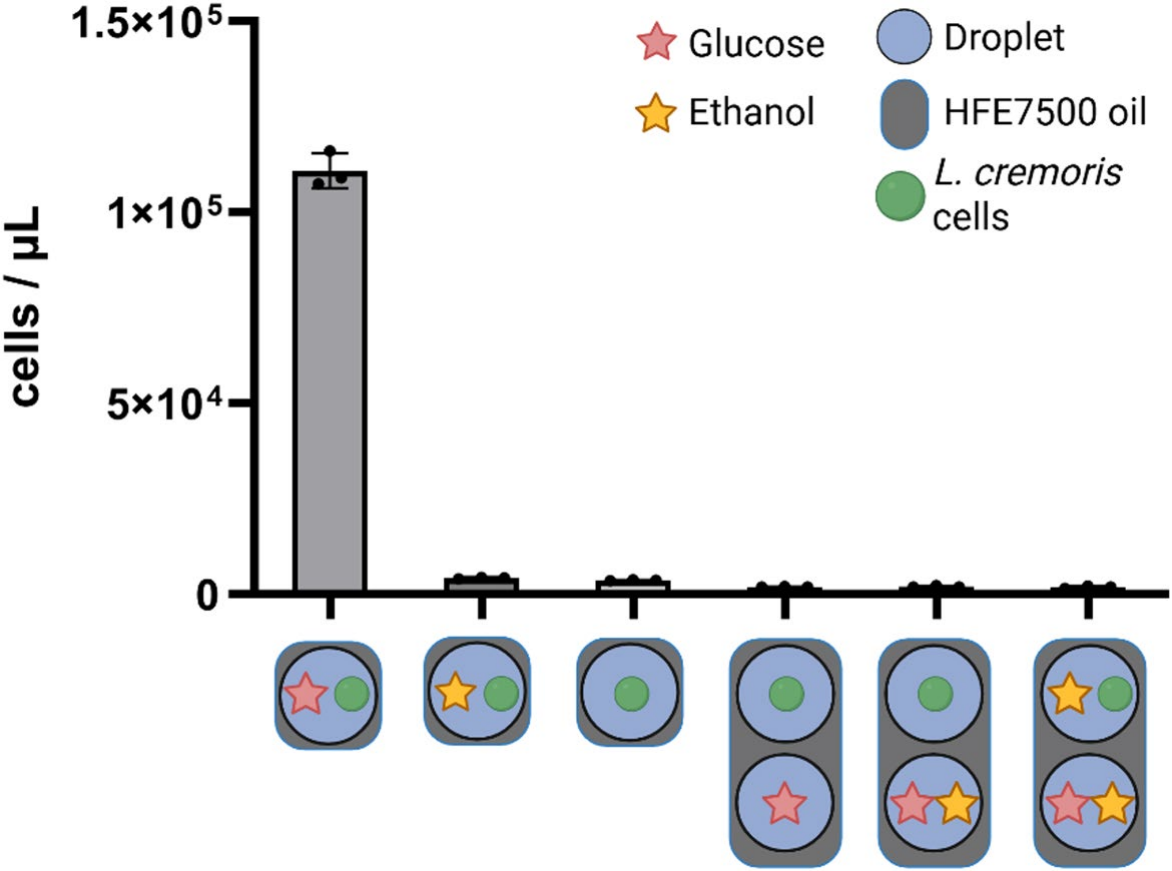
Ethanol as a co-solvent for glucose. Two types of emulsions were generated, containing CDMpc medium with either 2 wt% ethanol or 0.09 wt% glucose or no carbon source. Lactococcus cremoris was used as biosensor for molecule transfer. Since the organism can only grow on glucose, growth would indicate glucose transfer in the presence of ethanol. Growth was measured after 48 h incubation using flow cytometry. *n* = 3, mean ± SD. Figure was created in BioRender. Fecker, T. (2025): https://BioRender.com/iol885d.

Our results showed no restoration of growth of *L. cremoris* after the 2-day incubation period, regardless of the presence or absence of ethanol in the adjacent droplets, indicating that ethanol does not act as a co-solvent for glucose in this system (Fig. 3).

### Weak acids transfer and show uncoupling between droplets

**Fig. 4:**
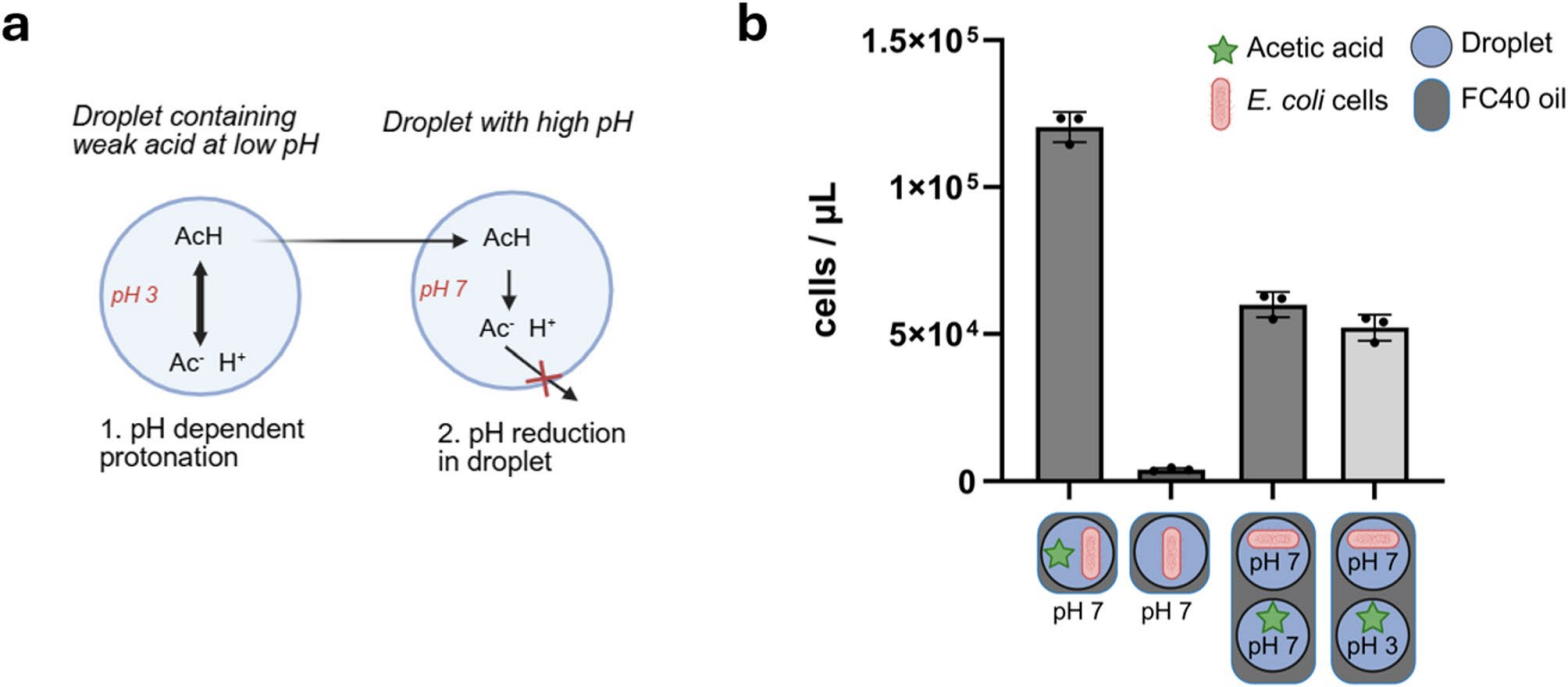
Transfer of acetic acid between droplets. (**a**) Schematics of weak acid decoupling. The weak acid is mostly protonated if the surrounding pH is below the pKa, which increases its hydrophobicity enabling it to pass through membranes. Once in the higher pH compartment, it deprotonates, reducing the pH inside the compartment. (**b**) Two types of emulsions were generated, containing M9 medium with either 0.1 wt% acetic acid or no carbon source, using E. coli as biosensor for molecule transfer. The organism can grow on acetic acid, so growth indicates the transfer of acetic acid between two droplets. *N* = 3, mean ± SD. Figure created in BioRender. Fecker, T (2025): https://BioRender.com/q670x9x.

To investigate the transfer of a third class of common molecules in biological experiments, weak acids, we selected acetic acid, a common metabolic product and carbon source. Since this acid shows pH-dependent protonation, it may also show differences in transfer between droplets, depending on the droplet pH (Fig. 4a).

To test a pH-dependent transfer of acetic acid, we used acetic acid-consuming *Escherichia coli* cells as biosensors. We prepared emulsions containing *E. coli* cells and mixed them with emulsions containing droplets with 0.1 wt% acetate at pH 3 or pH 7, at which the acid is either mostly protonated or deprotonated, respectively (pKa = 4.76). After 48 h of incubation, the cell concentration had increased irrespective of the pH of the acid-containing droplets (Fig. 4b), suggesting that, in emulsions, acetic acid can transfer between droplets, even at a pH at which it is mostly deprotonated.

**Fig. 5:**
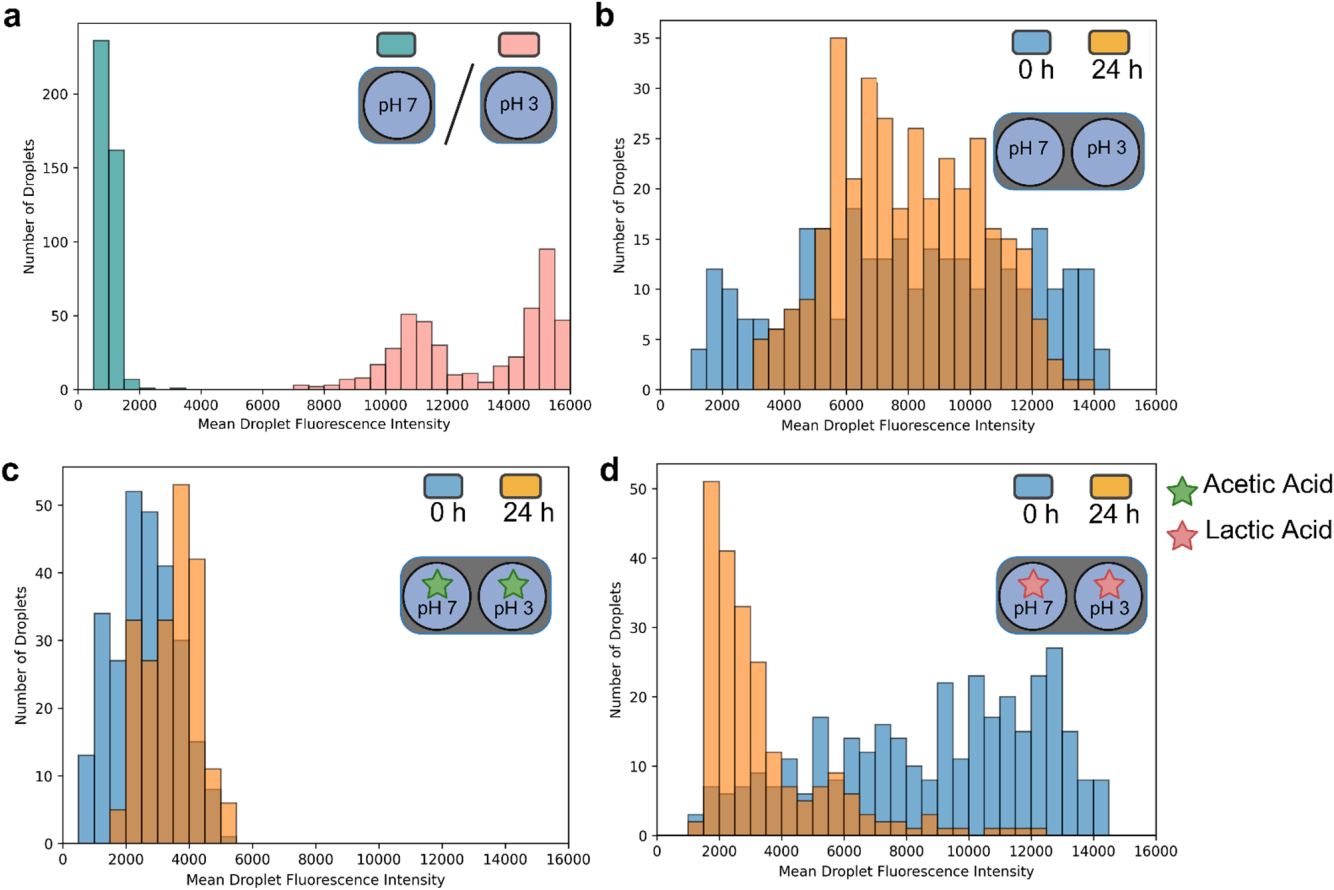
Quantification of droplet fluorescence intensity to assess weak-acid-mediated pH changes. Droplet fluorescence serves as a proxy for internal pH, based on the pH-sensitive emission of fluorophore 5,6-carboxyfluorescein. Each histogram represents the distribution of mean fluorescence intensities of droplets across several fluorescence microscopy images. (**a**) Fluorescence distribution of droplets containing M9 medium with 0.4 wt% acetate adjusted to either pH 3 or pH 7. b-d) Mixed emulsions containing droplets at pH 3 and pH 7, with or without added weak acids, were imaged immediately after mixing (t = 0 h) and after 24 h (t = 24 h). pH was adjusted to ensure mostly full protonation or deprotonation of weak acids. (**b**) Mixtures of droplets without added acids at pH 3 and pH 7 (M9 only). (**c**) Mixtures of droplets containing 0.4 wt% acetate at pH 3 and pH 7. (**d**) Mixtures of droplets containing 0.4 wt% lactate at pH 3 and pH 7. Figure created in BioRender. Fecker, T. (2025): https://BioRender.com/ciaxng7.

We hypothesized that acetic acid would preferentially transfer in the protonated form due to its higher hydrophobicity^29^ and then lower the pH in the droplets containing cells, resulting in a net transfer of protons among the droplets (Fig. 4a). To test this, we added the pH-dependent fluorophore 5,6-carboxyfluorescein to the emulsions, which exhibits green fluorescence above pH 5^30 ^. Using fluorescence microscopy and an image analysis pipeline (described in supplementary files, Sect. 5), we quantified the fluorescence intensity of individual droplets. As expected, droplets containing buffer at pH 7 exhibited strong green fluorescence, while those at pH 3 showed no fluorescence (Fig. 5a, Supplementary Figure S3).

Next, we mixed emulsions of droplets at pH 7 with droplets at pH 3 and measured fluorescence immediately and after 24 h. In the absence of weak acids, both droplet populations remained distinct over time, indicating stable pH conditions (Fig. 5b, Supplementary Figure S3). We observed a minor fluorescence shift in this system, suggesting slow pH equilibration, potentially via buffer components present in the medium.

When adding 0.4 wt% acetic acid to the pH 3 droplets, we observed an immediate decrease of fluorescence of the pH 7 droplets, which persisted after 24 h. This change indicated rapid transfer of protons facilitated by acetic acid (Fig. 5c). In contrast, the mixture with 0.4 wt% lactic acid initially maintained two distinct populations before converging to a single, low-intensity population after 24 h, suggesting slower transfer of lactate (Fig. 5d, Supplementary Figure S3). These results demonstrate that weak acids can influence the pH of neighboring droplets in the system, potentially impacting the growth of cells in neighboring droplets.

### Influence of oil type and surfactant on ethanol transfer

**Fig. 6:**
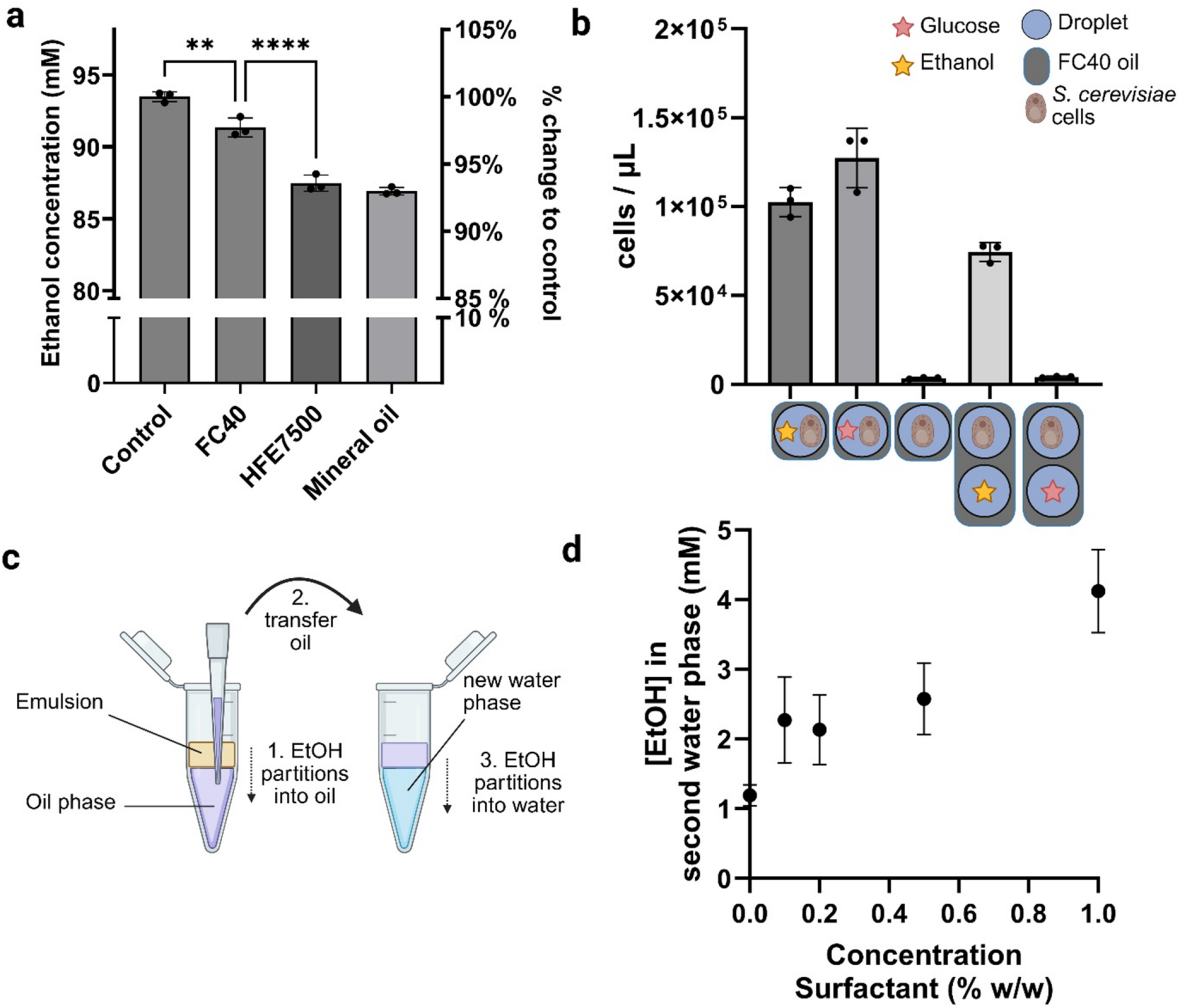
Effect of oil choice and surfactant on the transfer of ethanol. (**a**) Reduction of ethanol concentration in an aqueous phase when mixed with different oils at 1:9 water: oil ratio (avg ± SD, *n* = 3. Statistical test: multiple t.tests, unpaired, corrected with Holm-Šídák method (**: *p* = 0.0012, ****=*p* < 0.0001). (**b**) Transfer of ethanol in a biological experiment using the perfluorinated FC40 oil. Cell growth indicated ethanol transfer and a disruption of compartmentalization (avg ± SD, *n* = 3). (**c**) Experimental scheme to assess the partition coefficient of ethanol in oil. An emulsion is generated using a water phase (ethanol in dH_2_O) and an oil phase (HFE7500 with surfactant). After phase separation, the oil phase, now containing ethanol, is transferred to a fresh water phase. Ethanol partitions into the new water phase, and its concentration is quantified by HPLC. Figure created in BioRender. Fecker, T. d) Ethanol concentration in secondary water phase with varying surfactant concentration, as described in C) (avg ± SD, *n* = 3). Figure created in BioRender. Fecker, T. (2025): https://BioRender.com/2b2d7pt.

To investigate if the transfer of ethanol among microdroplets could be reduced by selecting another oil, we compared ethanol partitioning in FC40, HFE7500 and mineral oil. Unlike HFE7500, which contains a hydroxyl group, FC40 is perfluorinated, meaning that all CH groups are replaced by CF groups. The hydroxyl group in HFE7500 may interact with the hydroxyl group of ethanol, facilitating its solubility in the oil and thereby accelerating its transfer to other microdroplets. To test this, we mixed aqueous ethanol solutions with all three oils and measured the resulting ethanol partitioning in comparison to the water phase without oil. We chose a lower ethanol concentration than in Fig. 1 to reflect the range of ethanol concentrations found in biological experiments^23,24^. Despite being perfluorinated, ethanol still showed significant partitioning into FC40, however less than into HFE7500 (Fig. 6a).Mineral oil showed the highest ethanol partitioning of all tested oils, and it is likely that the biological outcome would therefore be similar or exacerbated compared to HFE7500.

To assess if the reduced ethanol partitioning into FC40 would be sufficient to prevent growth via ethanol transfer, we repeated the biosensor assay using FC40 as the oil phase. Similar to the HFE7500 experiment (Fig. 2), droplets containing *S. cerevisiae* cells were mixed with droplets containing a carbon source. When ethanol was present, cells grew, indicating ethanol transfer between droplets, while no growth was observed when glucose was used, confirming no glucose transfer (Fig. 6b). The results are consistent with those obtained with HFE7500, suggesting that using FC40 as the oil phase could not impede transfer-mediated growth despite its reduced ethanol partitioning.

We hypothesized that the ethanol transfer between microdroplets by direct partitioning into the oil phase would be enhanced in the presence of surfactant. Surfactants are required to stabilize the emulsion, but have been shown to increase the solubility of small molecules in the oil phase, and therefore exacerbate the transfer.

To test this, we generated emulsions containing ethanol in the aqueous phase with varying surfactant concentrations in the oil. After phase separation, we recovered the oil phase from the bottom and added it to a second water phase. Subsequently, we measured the ethanol concentration using HPLC after allowing the ethanol in the oil phase to partition into the water phase for 6 h (Fig. 6c). Results showed that the surfactant had a measurable influence on increasing ethanol partitioning (Fig. 6d).

These findings suggest that changing the oil or adjusting the surfactant concentration may not be sufficient to prevent ethanol transfer in microdroplet experiments, highlighting the challenges in maintaining compartmentalization in such systems.

## Discussion

We found that several molecules commonly used in biological high-throughput experiments can transfer between droplets, disrupting the intended compartmentalization. Most notably, short chain alcohols and weak acids were able to transfer, leading to unintended cell growth and pH changes in neighbouring droplets. Ethanol was able to directly partition into the oil phase across different oils and concentrations. The extent of partitioning into HFE7500 oil was comparable for two different concentrations (6.3 ± 0.3% in Fig. 1 versus 6.2 ± 1.2% in Fig. 6a), supporting the robustness of this phenomenon across a biologically relevant concentration range. In contrast, sugars like glucose did not transfer, as indicated by the absence of cell growth in other droplets. While previous studies have reported molecule transfer in fluorinated emulsions^14,16^, our results demonstrate the functional consequences for biological droplet assays in polydisperse systems.

The transfer behaviour of different molecules between droplets can be in part explained by their physicochemical properties. Short-chain alcohols such as ethanol are small, amphiphilic molecules with high water/octanol partition coefficients, allowing for transfer (logP, see Table 1). In comparison, sugars as well as amino acids, which have a low logP due to their zwitterion status at physiological pH, did not transfer, while the nucleotide uracil with a logP of −1.1 (Table 1), which is close to the logP of methanol, did transfer. Although logP is defined for hydrocarbon-based oils and may not directly translate to fluorinated oils^16^, previous studies have found a strong correlation between molecule hydrophobicity and transfer rate in droplet systems^14,16,18,19^. In a recent survey of molecules, logP was identified as the strongest predictor of molecule transfer^14^. Our findings are consistent with these results and support the use of hydrophobicity as a predictor of transfer susceptibility in microdroplet assays.

Weak acids, such as acetic acid, also showed inter-droplet transfer. When fully protonated, these molecules have logP values similar to short-chain alcohols, while also being small (< 100 g/mol) and amphiphilic (Table 1). We showed that protonated acetic acid could transfer from pH 3 droplets to pH 7 droplets, where it was consumed by growing organisms. This behaviour resembles weak acid uncoupling, in which protonated weak acids diffuse from a low-pH environment into cells, deprotonate in the higher-pH cytoplasm, and lower the intracellular pH^31^. Using a pH-sensitive fluorophore, we confirmed that the transfer not only resulted in a delivery of carbon to the droplets but also lowered the pH in neighbouring droplets, suggesting that droplet emulsions are susceptible to weak acid uncoupling at low pH (Fig. 5).

When deprotonated at neutral pH, weak acids become negatively charged and exhibit much lower partition coefficient values, comparable to glucose (Table 1, logD). Despite this, we still observed transfer and resulting growth in other droplets (Fig. 4). There is still a fraction of protonated acetic acid at this pH, which may have transferred, causing the cell growth in the neighbouring droplets. Conversely, a similar effect has been reported in water-in-oil-in-water emulsions, where transfer of hydrophilic molecules can occur via micelles or small aqueous compartments^21^. The amphiphilic nature of weak acids possibly enables interactions with surfactant molecules, which increases their solubility in the oil phase and mediates transfer between droplets via inverse micelles^19,20^.

Shuttling by inverse micelles might also exacerbate the transfer of small-chain alcohols. Such a dual mechanism has been reported for other molecules^14,32^. Consistent with this, we observed that ethanol transfer increased with rising surfactant concentration (Fig. 6d), indicating that micelle-mediated transport may exacerbate molecule transfer for small, amphiphilic molecules. Further experiments to test the effect of reverse micelle shuttling could elucidate the contribution of the different mechanisms more clearly, potentially leading to new methods to prevent transfer.

**Table 1:** Tested molecules and selected physicochemical properties. LogP refers to the total water/octanol partition coefficient of all molecular subspecies, logD refers to the partition coefficient of a subspecies at a certain condition, in this case at a pH of 7.4. logD values are only available for molecules that have a pK_a_.

Molecule	Molecular formula	Molecular weight (g/mol)	logD (pH 7.4) ^a^	logP ^b^	pK_a_
Ethanol	C_2_H_4_OH	46	-	−0.31	-
Methanol	CH_3_OH	32	-	−0.77	-
Acetic acid	C_2_H_4_O_2_	60	−2.9	−0.17	4.8^33 ^
Lactic acid	C_3_H_6_O_3_	90	−3.7	−0.72	3.8^34^
Xylose	C_5_H_10_O_5_	150	-	−2.5	-
Glucose	C_6_H_12_O_6_	180	-	−3.0	-
Glycerol	C_3_H_8_O_3_	92	-	−1.8	-
Uracil	C_4_H_4_N_2_O_2_	112	−0.87	−1.1	9.4^35^

Amino acids	Molecular formula	Molecular weight (g/mol)	logD (pH 7.4) ^c^	logP ^c^	pK_a_^b^
Leucine	C_6_H_13_NO_2_	131	−1.9	0.7 ^c^	2.4/9.6
Valine	C_5_H_11_NO_2_	117	−2.2	0.2 ^c^	2.4/9.6
Histidine	C_6_H_9_N_3_O_2_	155	−3.1	−1.3	1.8/6.0/9.2
Methionine	C_5_H_11_NO_2_S	149	−1.9	0.4	2.3/9.2

^a^ Extracted from ChEMBL^36^.

^b^ Extracted from PubChem^37^.

^c^ Calculated using ChemSpider’s ACD/Labs model.

The observed transfer of some common molecules can potentially affect biological experiments by disrupting the droplet compartmentalization, especially in setups that require precise control over nutrient availability, such as yield-based selection screens. Here, each droplet is supplied with a defined, limited amount of substrate, and cells that achieve high cell number yield per unit carbon are selected for^6,38^. If substrates or metabolic byproducts transfer between compartments, droplets containing fast-growing cells could become carbon sinks, allowing them to scavenge additional substrate from neighbouring droplets, and appearing with a higher yield^39^.

As an example for setups that might be affected, Rabbers et al. successfully evolved *E. coli* strains with improved yield on glucose and the overflow metabolite acetate^40^. Although the concentrations of these byproducts are typically low and diluted over the system, their leakage may still affect the producer cells, which lose carbon to the surrounding environment. Microdroplet systems have also been used to select strains for improved ethanol production^41,42^, a setup that could similarly be affected by inter-droplet transfer. Production of weak acids at low pH may unintentionally acidify adjacent droplets, potentially reducing cell viability.

Controlled metabolite transfer between droplets could potentially be exploited intentionally to enable new experimental designs. For example, methanol – a molecule we showed to diffuse readily into fluorinated oil (Fig. 1) – is a well-established inducer of gene expression in methylotrophic yeasts such as *Komagataella phaffi* (*Pichia pastoris*)^43^. A mutant library could be encapsulated in droplets and grown under non-inducing conditions, after which methanol is administered into the oil phase or into reservoir droplets to diffuse into the target droplets and induce targeted gene expression.

A similar strategy has been demonstrated for inducers such as IPTG or AHL, which successfully diffused between droplets to induce GFP expression in a microfluidic setup^44^. However, this approach would require careful tuning of droplet spacing and methanol transfer rate to achieve desired delivery. More broadly, small-molecule diffusion could serve as an in situ substrate delivery system, effectively simulating a miniaturized fed-batch system within an emulsion^45^. This opens new possibilities in high-throughput screening setups that are currently static.

To mitigate unintended molecule transfer between droplets, several strategies have been previously explored, among others the use of additives to reduce molecule transfer. For example, bovine serum albumin (BSA) has been shown to interact with transferring fluorophores, retaining them in the water phase^12,17,45^. Similarly, novel surfactants have been developed and shown to reduce molecule transfer^46,47^. Another strategy replaces conventional surfactants with solid particles to form Pickering emulsions. This prevents reverse micelle formation entirely and has been shown to enhance the retention of the fluorophore resorufin^32^.

However, since these alternative surfactants may reduce transfer mediated by micelle formation, they will not be able to inhibit the transfer of molecules that partition directly into the oil phase, such as alcohols. While it has been suggested that Pickering emulsion may form a barrier at the droplet interface to prevent partitioning, such emulsions are often not fully covered, leaving sufficient space for molecules to enter the oil phase^32,48^ Similarly, the effect observed with bovine serum albumin has only been shown to apply only to large molecules like fluorophores and cannot be translated to small molecules^17^. We therefore expect that the suggested remedies will not prevent the transfer of directly partitioning molecules. However, it remains to be shown if they can present a solution for molecules that do not partition directly, but transfer nonetheless. Future work systematically testing these strategies on different molecules could provide insight about their utility in preventing transfer.

Based on our work, we recommend that researchers carefully consider their molecules when designing droplet-based biological assays. Specifically, we encourage experimental validation of both compartmentalization and its biological effects, especially when working with small, amphiphilic molecules. When strict compartmentalization is required, primary substrates with low logP values (< 1.5), such as sugars and glycerol, are preferable. If a substrate is shown to transfer, researchers should test if the molecules directly partitions into the oil. In such cases, a fully perfluorinated oil (e.g. FC-40) might reduce transfer sufficiently. If micelle shuttling is suspected, using transfer-resistant surfactants or nanoparticles can help eliminate transfer. For metabolic products prone to transfer, reducing the cell-to-droplet ratio can dilute transferred molecules, lowering the effect on other cell-containing droplets. While no single strategy is likely to fully eliminate molecule transfer, careful consideration of the experimental design may significantly reduce transfer and ensure robust experimental outcomes.

To our knowledge, this is the first study to demonstrate the biological effect of the transfer of common substrates and (by)products encountered in high-throughput screening experiments. The biosensor assay can be adapted to test other molecules, provided that a consuming strain is identified for the chosen molecule. While our approach offers a simple and accessible framework to assess the effect of molecule transfer, it has limitations. The setup was only tested on a limited range of molecules, and future studies might include more nucleic acids, or other biologically relevant molecules. Additionally, while most studies use monodisperse droplets generated in microfluidic systems, we tested polydisperse emulsions generated by vortexing. The size variation in our setup might exacerbate the transfer by additional systemic effects, such as Ostwald ripening. We showed that droplet ageing cannot explain the difference in transfer between different molecules and ageing did not induce transfer of glucose (Supplementary Sect. 2, Figure S2). This suggests a minor role of Ostwald ripening for transfer, but a more rigorous study of droplet ageing could elucidate the effects introduced by polydisperse emulsions. We therefore cannot readily extend the generated data to the more commonly used monodisperse emulsions. Lastly, since the scope of this study was the biological effect of molecule transfer, we did not do more rigorous testing of the mechanisms governing transfer. Such studies have been performed previously and directly led to the development of novel surfactants limiting transfer^14,20,32^. This study demonstrates that molecule transfer can significantly affect the experimental outcome of biological high-throughput experiments with potentially broad implications for mutant selection or growth assays using droplet systems. By giving practical recommendations for researchers, we encourage researchers working with droplet setups to carefully consider the potential of substrate or product transfer and their effects on neighbouring environments and to validate compartmentalization of the molecule in question.

## Methods

### Microbial strains and growth conditions

*Saccharomyces cerevisiae* CEN.PK113-7D and CEN.PK113-7D were grown on high concentration synthetic medium (HCSM), containing per litre: 10 g KH_2_PO_4_, 5 g MgSO_4_·7H2O, 6.6 g K_2_SO_4_, 9 g CH_4_N_2_O (urea), trace elements and vitamins^53^. When required, it was supplemented with 150 mg/L uracil^54^. *Escherichia coli*MG1655 was grown on M9 minimal medium, described by Maniatis et al.^55^. *Lactococcus cremoris* MG1363 was grown on CDMpc, described by Price et al.^56^. Glucose, ethanol, and acetic acid were used as carbon sources, in the concentrations as indicated. When required, single amino acids were left out of the final medium to test their transfer.

**Table 2:** Strains used in this study.

Strain	Description	Genotype	References
S. cerevisiae CEN.PK113-7D	Common Laboratory strain (wildtype)	*MATa MAL2-8c SUC*2	^27,49 ^
S. cerevisiae CEN.PK113-5D	CEN.PK113-7D (uracil auxotroph)	CEN.PK113-7D *ura3-52*	^27 ^
E. coli MG1655	K-12 wildtype strain	F^–^ λ^–^ ilvG^–^ *rfb*–50 *rph*–1	^50 ^
L. cremoris MG1363	Common laboratory strain (wildtype)	-	^51,52 ^

The *S. cerevisiae* strains were pre-cultured in HCSM + 2 wt% glucose at 30 ˚C in 100 mL round-bottom shake flasks in a shaking incubator. The *E. coli* MG1655 strain was precultured in M9 + 0.2 wt% glucose in aerated, 50 mL Greiner tubes at 37 ˚C in a shaking incubator. The *L. cremoris* MG1363 strain was pre-cultured in CDMpc + 0.09 wt% glucose in 50 mL Greiner tubes at 30 ˚C in a standing incubator. Freezer stocks were prepared by growing the strains in the indicated medium with appropriate carbon source and storing the stationary culture (exponentially growing for *S. cerevisiae*) at −80˚C with 20 vol% glycerol (*S. cerevisiae* with 30 vol%).

### Quantification of metabolite partitioning

To quantify the extent of metabolite partitioning, metabolites were diluted in 1x phosphate buffered saline (PBS) and their concentration was determined using high-performance liquid chromatography (HPLC). The concentration of each molecule was chosen according to the resolution of the HPLC. Hundred microliters of each metabolite solution was added to 900 µL HFE7500 oil (3 M, Two Harbors, MN, USA), FC40 oil (3 M, Two Harbors, MN, USA) or mineral oil (Merck, Darmstadt, Germany) and vortexed (Vortex-Genie 2, Scientific Industries inc, Bohemia, NY, USA) on maximum speed for 5 min. Afterwards, the concentration of the molecule in the liquid phase was determined using HPLC. Statistical analysis was performed on the whole dataset, one-tailed t-tests, assuming not equal standard deviation among the samples. One-tailed was chosen to indicate that only the partitioning of the molecule from water phase to oil was tested.

### Generation and quantification of cell growth in microdroplets

Cell-containing emulsions were generated according to^6^, with slight alterations. Cells were pre-cultured to stationary phase and washed once with 1x PBS. Afterwards, the cell concentration was determined using flow cytometry (Accuri C6, BD Biosciences, San Jose, CA, USA). Based on the measured concentration, the cells were diluted to a final concentration of 3.0·10^7^ cells/mL using the corresponding growth medium and carbon source. From this aqueous phase, 300 µL was added to 700 µL of either HFE7500 + 0.2 vol% PicoSurf 1 surfactant (Sphere Fluidics, Cambridge, UK) or FC40 oil + 0.4 vol% PicoSurf 1 surfactant. Afterwards, emulsions were generated by agitating for 5 min at 2500 rpm using a multi-tube vortexer (DVX-2500, VWR, West Chester, PA, USA) and placed on ice. The generated droplets had a representative volume ± SEM of 37.6 ± 6.9 pL (HFE7500) and 36.9 ± 6.5 pL (FC40) (Supplementary Sect. 1). With this droplet size and the above mentioned cell concentration of 3.0·10^7^ cells/mL, there will be on average 1 cell per droplet.

The prepared emulsions were covered with parafilm to ensure sufficient oxygen transfer and incubated in a standing incubator at 30 ˚C (*S. cerevisiae* and *L. cremoris*) or 37 ˚C (*E. coli*) for 72 h (*S. cerevisiae* and *L. cremoris*) or 48 h (*E. coli*). After incubation, the emulsions were added to a 15 mL Greiner tube, and broken by addition of 1 mL PBS and 1 mL perfluorooctanol (PFO, Alfa Aesar, Ward Hill, MA, USA) with gentle mixing. The resulting water layer was transferred to an Eppendorf tube and the cell concentration was measured using flow cytometry.

### Visualization of pH transfer in microdroplets

To visualize pH in microdroplets using microscopy, a 5(6)-carboxyfluorescein (Merck, Darmstadt, Germany) solution was prepared by adding 4 mg 5(6)-carboxyfluorescein to 100 µL of 100% ethanol and then adding 900 µL of demineralized water. This was added to 100 mL of M9 for a final concentration of 40 ppm carboxyfluorescein. Solutions of M9 with the indicated carbon source and pH (pH set with either 2.5 M HCl or 5 M NaOH) were used to generate emulsions as indicated above. The resulting emulsions were mixed, if required, and imaged immediately, as well as after incubation for 24 h at 30 ˚C in a standing incubator, using fluorescence microscopy (Zeiss Imager Z1, Zeiss, Jena, Germany) at 200x magnification. The fluorescence of individual droplets was analyzed using a custom Python script (supplementary Sect. 4).

### Estimation of ethanol partitioning in emulsions

To assess the effect of surfactant concentration on ethanol partitioning, 300 µL of aqueous phase containing PBS with 100 mM ethanol was added to 700 µL of HFE7500 oil + PicoSurf 1 surfactant at the indicated concentration. Emulsions were generated as described above and incubated down at room temperature for 16 h to allow for settling of the microdroplets at the top. From the emulsions, 400 µL of the oil was removed from the bottom and added to 100 µL PBS without agitation. The solution was incubated at room temperature for 6 h, and the resulting ethanol concentration in the PBS was measured using HPLC.

## Supplementary Information

Below is the link to the electronic supplementary material.

Supplementary Material 1

## Data Availability

The authors declare that the data supporting the findings of this study are available within the paper and its Supplementary Information files. Should any raw data files be needed in another format they are available from the corresponding author upon reasonable request.
