# Supplementary material for: The effect of substrate and product transfer between polydisperse microdroplets on biological high-throughput screens
Source: Sci Rep. 2026 Feb 2;16:4318. doi: 10.1038/s41598-025-25878-5 (PMC12865034; doi:10.1038/s41598-025-25878-5)
Supplement: Supplementary file 1 — Supplementary Material 1 [file 41598_2025_25878_MOESM1_ESM.pdf]

Supplementary information

The effect of substrate and product transfer between polydisperse microdroplets on biological high-throughput screens

T Fecker[1], T de Kanter[1], RJ van Tatenhove-Pel[1]*

[1]Department of Biotechnology, Delft University of Technology, van der Maasweg 9, 2629 HZ, Delft, The Netherlands

**Corresponding author:* R.J.vanTatenhove-Pel@tudelft.nl

Section 1: Droplet size estimation pipeline

To estimate the average size of generated microdroplets, we followed the protocol described in the Materials and Methods section. We imaged the resulting polydisperse microdroplets at 200x magnification using nine images per emulsion (Fig. S1a). These images were then processed with a custom imageJ pipeline, that applied a watershed algorithm to segment the droplets (Fig. S1b). Very small droplets (< 2 μm) and droplets from the border of the picture were excluded from the analysis.

The diameters of the segmented droplets were extracted and pooled over all images. Since the vortex-based droplet generation method generates polydisperse emulsions, a wide range of droplet sizes was observed. Although smaller microdroplets (< 20 μm) are more abundant (Fig. S1c), due to a low volume of these microdroplets, they contribute less to the total volume of the aqueous phase (Fig. S1d). In contrast, larger droplets account for a greater share of the volume. To calculate the representative droplet size, we analysed droplet size distribution normalized by the volume fraction. Specifically, for each emulsion, we identified the five droplet size bins contributing the most to the total volume and averaged these sizes. This value was taken as the volume-weighted average droplet size. We repeated this process across multiple emulsions to obtain an estimate of the variation in droplet size (Standard error of the mean, SEM). The resulting representative droplet size for the volume-weighted size distributions is 37.6 ± 6.9 pL (40.6 ± 2.7 μm diameter)

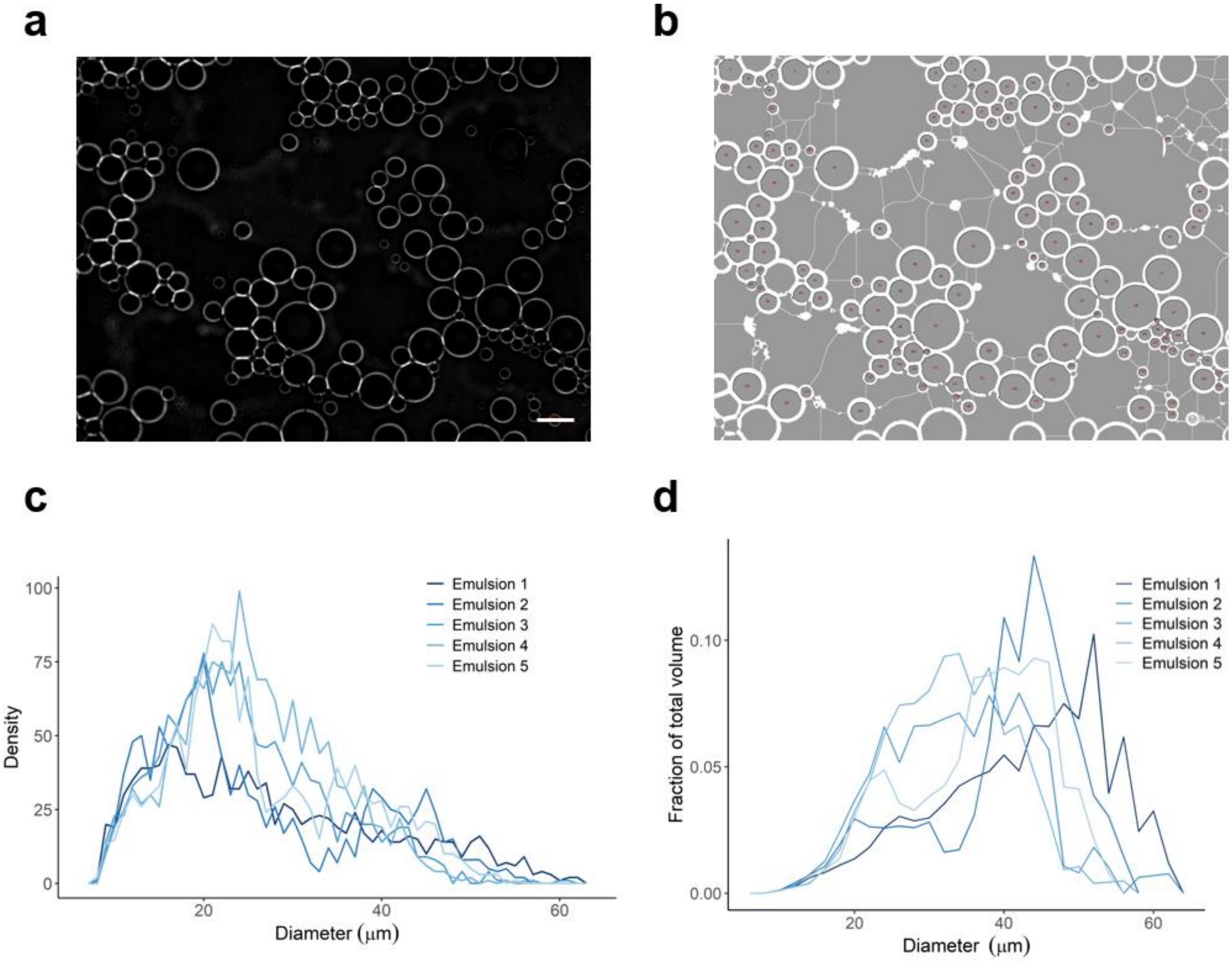

Figure S1: Droplet size analysis pipeline. a) Microscope image of microdroplets. Scale bar: 50 μm. b) Droplets segmented using watershed. c) Histogram of droplet sizes in five emulsions d) Diameter distribution normalized by total volume. The top 5 most occurring diameter bins of each emulsion are averaged to calculate the representative droplet size.

Section 2: Differences in droplet generation between substrates

Our system uses bulk emulsions, that display a wider size distribution than monodisperse emulsions generated with microfluidic devices. To test if the composition of the aqueous phase influences droplet size or ageing for this method, we prepared emulsions containing either 2 wt% ethanol or 8 wt% glucose.

We analyzed the distribution of droplet sizes using the ImageJ particle size estimation pipeline described in Supplementary section 1. Both the average droplet size (Fig. S2a) and the size distribution (Fig. S2b) were similar between glucose and ethanol containing microdroplets, indicating that the selective transfer ethanol cannot be explained by differences in microdroplet generation.

Next, we explored whether ethanol might influence droplet ageing over time, explaining its selective transfer. Specifically, we were interested if the average droplet size would change with either molecule, or if the number of small droplets, potentially acting as molecular shuttles, would change between the different emulsions over time. Neither the number of small droplets (Fig. S2c), nor the size (Fig. S2D) showed a difference between both different emulsions that could explain the selective transfer of ethanol, suggesting that the transfer of ethanol is more likely related to its molecular properties rather than a property of the emulsion system.

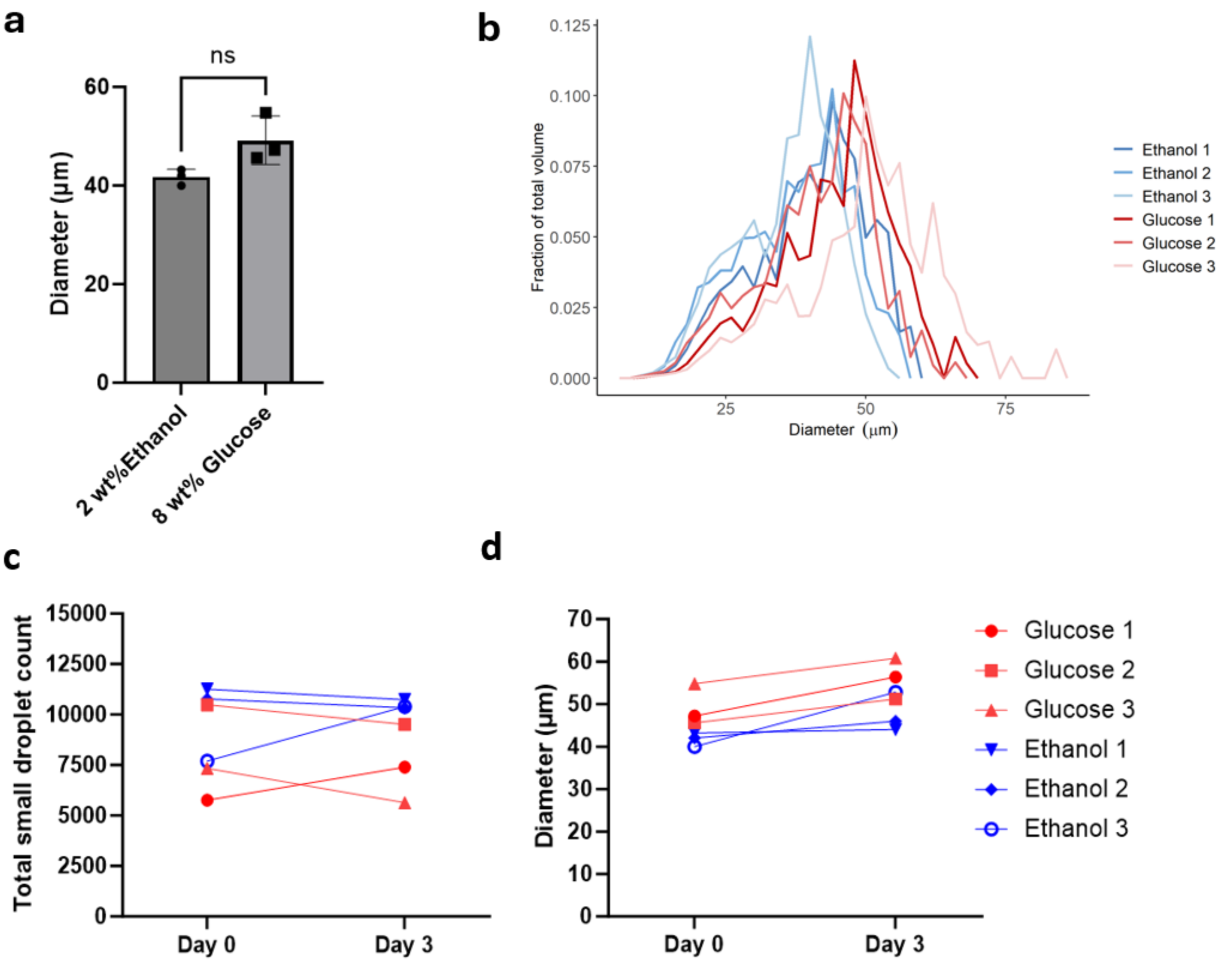

Figure S2 : Size and ageing behaviour of emulsions containing ethanol or glucose. a) Average size of microdroplets. Microscope images are taken of each emulsion and the imaged droplets are measured for size as a fraction of total volume. Three such emulsions are prepared and characterized (bars: average ± SD). Statistical test: student t.test, unpaired, two-tailed, α=0.05 ns: p=0.067. b) Resulting distribution of all assessed droplets within each emulsion as a fraction of total volume. c) Number of small droplets (< 2 µm) per picture upon generation and after three days. The number was assessed with image analysis from microscope images. d) Average droplet size upon emulsion generation and after 3 days.

Section 3: Imaging stained droplets

To support the histograms shown in figure 5, we include representative fluorescence microscopy images of the same droplet emulsions used to generate the histograms (Fig. S3).

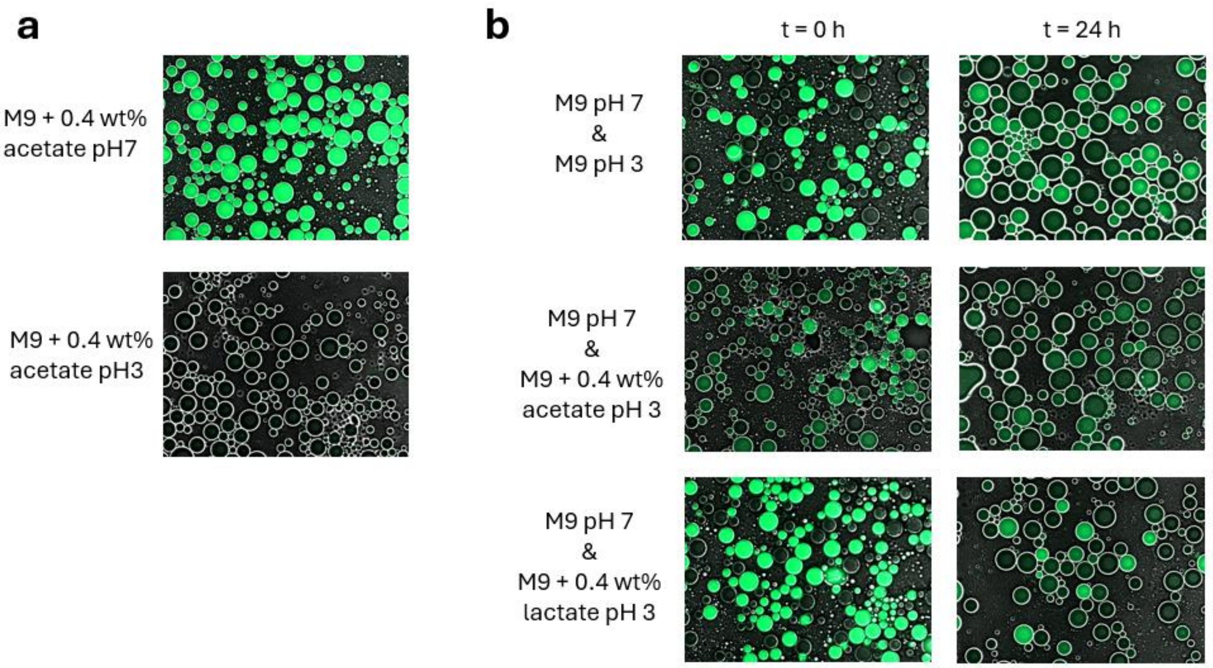

Figure S3: Representative fluorescence microscopy images corresponding to the droplet fluorescence histograms shown in Figure 5. Droplets contained medium and the pH-dependent fluorophore 5,6-Carboxyfluorescein that shows fluorescence at pH >5 were generated and imaged. a) Representative images of single emulsions of M9 at either pH 7 or pH 3. b) mixtures of emulsions containing M9 pH7 + M9 pH3 and, if applicable, a weak acid. Representative images at t=0 h and t=24h.

Section 4: Segmentation of stained droplet populations

To quantify the droplet fluorescence, we used an image analysis pipeline, based on droplet segmentation. We obtained images by fluorescence microscopy, labelled them using the watershed algorithm on the fluorescence channel and then calculated the mean fluorescence for all droplets in each image. For each condition, we analysed >250 droplets. The segmentation was manually reviewed and corrected using Napari [1].

References:

1. Chiu, C. & Clack, N. napari : a Python Multi-Dimensional Image Viewer Platform for the Research Community. *Microsc. Microanal.* **28**, 1576–1577 (2022).
